# Frailty in peritoneal dialysis patients: a scoping review

**DOI:** 10.3389/fmed.2026.1906681

**Published:** 2026-08-20

**Authors:** Leyao Zheng, Xinlan Hu, Zhongci Zhong, Xiaoqiao Qiu, Jin Wang, Lijuan Dong

**Affiliations:** 1 Zhongshan Hospital of Traditional Chinese Medicine Affiliated to Guangzhou University of Chinese Medicine, Department of Internal Medicine IV, Zhongshan, Guangdong, China; 2 School of Nursing (Nursing School of Smart Healthcare Industry), Henan University of Chinese Medicine, Zhengzhou, Henan, China

**Keywords:** frailty, geriatric nursing, nursing, peritoneal dialysis, scoping review

## Abstract

**Objective:**

To conduct a scoping review of research on frailty in peritoneal dialysis (PD) patients, providing a basis for future studies.

**Methods:**

Following the scoping review reporting guidelines, a systematic search of domestic and international databases was performed for studies on frailty in PD patients from inception to April 10, 2026. Two researchers independently screened the literature and extracted data.

**Results:**

A total of 29 studies were finally included, of which 28 reported the incidence of frailty in PD patients, ranging from 7.3% to 83%, showing considerable variation across different studies and assessment tools. Frailty was influenced by multidimensional factors, including sociodemographic characteristics, disease-related variables, physical function and body composition indicators, and psychological-behavioral factors. The main assessment tools used were the Fried Frailty Phenotype Scale and the Clinical Frailty Scale (CFS). However, there is a lack of assessment tools specifically tailored to the disease characteristics of PD patients.

**Conclusion:**

The prevalence of frailty in PD patients varies considerably and is affected by multiple factors. Existing assessment tools require further optimization.

## 1 Introduction

Peritoneal dialysis (PD) is a home-based renal replacement therapy and one of the primary treatment modalities for end-stage renal disease (ESRD). Frailty is a geriatric syndrome characterized by a decline in physiological reserve and multisystem dysregulation (1), it is primarily characterized by reduced strength and impaired physiological function. Studies have shown that the prevalence of frailty among PD patients in China ranges from 69.4% to 72.7% (2, 3), moreover, frailty exhibits a trend toward younger onset in PD patients, with a prevalence of 47.3% even among those aged <65 years (4). Frailty is an independent predictor of poor prognosis and increased mortality in dialysis patients, and may largely contribute to the increased complexity of nursing care for this population (5). Prompt prevention and intervention targeting frailty may decrease the occurrence of adverse events, including falls, infections, extended hospitalizations, and death, among affected patients (6). A comprehensive review of the current status, assessment tools, and influencing factors of frailty in PD patients is of great significance for objectively and accurately evaluating the health status of PD patients, as well as for predicting their quality of life and survival prognosis. To date, research on frailty among PD patients has been characterized by a paucity of systematic synthesis, notable heterogeneity in the assessment tools employed, and marked discrepancies in the reported prevalence across studies. Moreover, the identification of contributing factors has been relatively dispersed, and there is a paucity of systematic analysis encompassing multidimensional domains such as sociodemographic characteristics, disease-related variables, psychological and behavioral factors, and body composition with functional parameters. Accordingly, following the scoping review methodology proposed by Arksey et al. (7), this study provides a comprehensive overview of the prevalence, assessment tools, and determinants of frailty in PD patients, aiming to inform targeted clinical prevention and intervention strategies to enhance patient prognosis. This study has been publicly registered on the OSF platform in accordance with the recommendations of the Joanna Briggs Institute (JBI) for Evidence-Based Healthcare (8), with the registration number 10.17605/OSF.IO/FG7ZK. The registration protocol can beaccessed via the following link: https://osf.io/fg7zk?view_only=b0bd146397b445808984d32bad081bc8.

## 2 Materials and methods

### 2.1 Identifying the research question

Based on the literature review, the following research questions were identified: (1) What is the current status of frailty in PD patients? (2) What assessment tools are available for evaluating frailty in PD patients? (3) What are the influencing factors of frailty in PD patients?

### 2.2 Inclusion and exclusion criteria

In accordance with the PCC (Population-Concept-Context) framework (9), the eligibility criteria for literature inclusion and exclusion were defined. The inclusion criteria were as follows: Participants (P) were PD patients; The concept (C) was defined as frailty. As stated in the relevant expert consensus (10), frailty represents a multidimensional construct that includes, but is not limited to, physical frailty, social frailty, cognitive frailty, and psychological frailty; The context (C) was the location of patients, including healthcare institutions, communities, and home settings. Exclusion criteria: Studies not published in Chinese or English; Full text not available; Review articles, systematic reviews, conference papers, case reports, study protocols, or other non-original research; Duplicate publications.

### 2.3 Search strategy

A systematic search was conducted in the China National Knowledge Infrastructure (CNKI), Chinese Biomedical Literature Database (CBM), VIP Database (VIP), Wanfang Database, PubMed, CINAHL, Web of Science, Cochrane Library, and Embase. The search period covered from the inception of each database to April 10, 2026. A combination of subject headings (MeSH terms) and free-text words was used as the search strategy. English search terms included “Peritoneal Dialysis” “Dialysis” “End-Stage Renal Disease” “Frailty” “Debility” “Frailty Syndrome” “Social frailty” “Physical Frailty” “Psychological Frailty” “Cognitive frailty”. Additionally, the reference lists of relevant studies were manually reviewed. Using PubMed as an example, the search strategy for English databases is presented in Table 1. The search strategies for the remaining Chinese databases are provided in Supplementary Table 1.

**Table 1:** Search strategy used in PubMed.

Step	Search query
#1	“Peritoneal Dialysis”[Mesh]
#2	((Dialyses, Peritoneal [Title/Abstract]) OR (Dialysis, Peritoneal[Title/Abstract])) OR (Peritoneal Dialyses [Title/Abstract])
#3	“Dialysis”[Mesh]
#4	Dialyses[Title/Abstract]
#5	“Kidney Failure, Chronic”[Mesh]
#6	((((((Renal Failure, Chronic[Title/Abstract]) OR (Chronic Renal Failure[Title/Abstract])) OR (End–Stage Kidney Disease[Title/Abstract])) OR (Disease, End–Stage Kidney[Title/Abstract])) OR (End Stage Kidney Disease[Title/Abstract])) OR (Kidney Disease, End–Stage[Title/Abstract])) OR (ESRD[Title/Abstract])
#7	#1 OR #2 OR #3 OR #4 OR #5 OR #6
#8	“Frailty”[Mesh]
#9	((((Frailties[Title/Abstract]) OR (Frailness[Title/Abstract])) OR (Frailty Syndrome[Title/Abstract])) OR (Debility[Title/Abstract])) OR (Debilities[Title/Abstract])
#10	(((Cognitive frailty[Title/Abstract]) OR (Social frailty[Title/Abstract])) OR (Physical Frailty[Title/Abstract])) OR (Psychological Frailty[Title/Abstract])
#11	#8 OR #9 OR #10
#12	#7 AND #11

This table presents the complete search strategy for retrieving literature on “peritoneal dialysis” and “frailty” in the PubMed database.

### 2.4 Literature screening and data extraction

Import the retrieved literature into End Note X9 software, remove duplicate references, then two researchers trained in evidence-based methods independently conduct the initial screening according to inclusion and exclusion criteria, followed by a full-text review for rescreening. If there is a disagreement, it is discussed and decided together with a third researcher. The extracted content includes the first author, publication year, country, sample size, study type, assessment tools, frailty score/incidence of PD patients, influencing factors, etc.

### 2.5 Collate, summarize, and report the results

This study employed a narrative synthesis approach to classify and summarize the extracted literature information. According to the five core dimensions of the research questions, the findings of the included studies were categorized as follows: (1) current status of frailty in PD patients; (2) influencing factors (classified into individual factors, disease-related factors, physical function and body composition indicators, and psycho-behavioral factors); (3) assessment tools (categorized and summarized by instrument name, developer and year, items, score range, Validation Status in PD/CKD, administration time in minutes, advantages, and limitations in the PD context). The findings are presented using a combination of tables and narrative descriptions. Tables are used to present the basic characteristics of the included studies (Table 2), characteristics of the assessment tools (Table 3), and results of methodological quality evaluations (Supplementary Table 2); the narrative section provides a systematic summary and interpretation of the tabulated content. The reporting of this scoping review strictly adheres to all item requirements of the PRISMA-ScR checklist, including background and purpose, eligibility criteria, sources of evidence, data extraction methods, results, and conclusions linked to the research questions and objectives. In addition, the Discussion section explicitly identifies the limitations of existing studies (e.g., predominance of cross-sectional designs, high heterogeneity in reported frailty prevalence among PD patients, and geographical concentration of included studies in China and South Korea) as well as future research directions (e.g., developing frailty assessment tools specifically validated for PD patients, exploring the underlying mechanisms of frailty in this population, and distinguishing modifiable from non-modifiable factors associated with frailty in PD patients).

**Table 2:** Basic characteristics of the included literature (*n* = 29).

References	Country	Sample size	Study design	Frailty tool	Prevalence/ score	Key findings
Chan et al. (2)	China	267	Prospective cohort study	CFS	72.7%	Baseline prevalence reported
Ng et al. (3)	China	193	Cross–sectional study	FI	69.4%	Overall prevalence only
Peng et al. (11)	China	147	Cross–sectional study	HALFT	20.4%	Overall prevalence only
Wang et al. (12)	China	275	Cross–sectional study	TFIA	28.0%	Overall prevalence only
Xie et al. (13)	China	52	Cross–sectional study	CHS		Prevalence not clearly stated
Yao et al. (14)	China	94	Cross–sectional study	CFS	Frailty prevalence:75.5%	Both physical and cognitive frailty assessed
				MMSE	Cognitive frailty prevalence :27.7%	
Yi (15)	China	138	Cross–sectional study	HALFT	29.7%	Overall prevalence only
Zhang et al. (16)	China	205	Cross–sectional study	Fried Frailty Phenotype Scale	24.3%	Overall prevalence only
Li et al. (17)	China	246	Cross–sectional study	Fried Frailty Phenotype Scale	35.0%	Overall prevalence only
Zhu et al. (18)	China	142	Cross–sectional study	CFS	50.7%	Overall prevalence only
Hu et al. (19)	China	164	Cross–sectional study	Fried Frailty Phenotype Scale	63.4%	Overall prevalence only
Zhu et al. (20)	China	58	Cross–sectional study	Barthel Index	74.1%	Barthel used as proxy for frailty (functional dependence)
Chen et al. (21)	China	528	Cross–sectional study	CFS	24.1%	Overall prevalence only
Du et al. (22)	China	420	Cross–sectional study	FRAIL	12.6%	Overall prevalence only
Meng et al. (23)	China	85	Cross–sectional study	Fried Frailty Phenotype Scale	40.0%	Overall prevalence only
Davenport (24)	United Kingdom	573	Cross–sectional study	CFS	7.3%	Overall prevalence only
Kamijo et al. (25)	Japan	119	Prospective cohort study	CFS	10.9%	Overall prevalence only
Mansur et al. (26)	Portugal	23	Cross–sectional study	Fried Frailty Phenotype Scale	47.8%	Overall prevalence only
Chen et al. (27)	China	337	Prospective cohort study	FI, Fried Frailty Phenotype Scale	76.6%	Prevalence varied by assessment tool
Liu et al. (28)	China	123	Cross–sectional study	FRAIL	43.9%	Overall prevalence only
Piedade et al. (29)	Portugal	74	Retrospective cohort study	EFS	71.6%	Overall prevalence only
Torres et al. (30)	Chile	39	Cross–sectional study	CFS	25.6%	Overall prevalence only
Chan et al. (31)	China	267	Cohort study	FQ	58.8%	Overall prevalence only
Lee et al. (32)	South Korea	403	Cohort study	Fried Frailty Phenotype Scale	34.8%	Overall prevalence only
Chun–Kau et al. (33)	China	209	Cross–sectional study	FI	60.8%	Overall prevalence only
Cao et al. (34)	China	213	Mixed–methods study	Fried Frailty Phenotype Scale	12.2%	Overall prevalence only
Yi et al. (35)	China	784	Cohort study	CFS	27.6%	Overall prevalence only
Peralta–Flore et al. (36)	Mexico	106	Cross–sectional study	SPPB	83.0%	Overall prevalence only
Kobayashi et al. (37)	Japan	58	Cohort study	OFI−8	29.4%	Overall prevalence only

**Table 3:** Frailty assessment tools used in the included studies.

Assessment tool	Developer (Year)	Items	Score range & cut–off	Validation status in PD/CKD	Admin. time (min)	Advantages	Limitations (in PD context)
Fried Frailty Phenotype	Fried et al. (2001)	5	0–5; Frailty: ≥3	Yes (validated in hemodialysis and early CKD; limited PD–specific data)	10–15	Gold standard; objective measurements (grip strength, gait speed)	Requires physical performance tests; not feasible for bedridden or severely edematous PD patients
CFS (Clinical Frailty Scale)	Rockwood et al. (2009)	9	1–9; Frailty: ≥5	Yes (validated in general CKD; widely used in PD cohorts)	2–5	Quick, no equipment needed; relies on clinical judgement; predicts mortality in PD	Subjective; inter–rater variability; lacks physical performance components
TFI (Tilburg Frailty Indicator)	Gobbens et al. (2010)	15	0–15; Frailty: ≥5	Limited (validated in community elderly; not specifically in PD/CKD)	5–10	Multidimensional (physical, psychological, social); patient–reported	Time–consuming; some items (e.g., weight loss) confounded by fluid overload in PD
FRAIL Scale	Morley et al. (2012)	5	0–5; Frailty: ≥3	Emerging (few studies in CKD; initial validation in dialysis)	2–3	Very quick; simple questionnaire; no equipment	Fatigue and weight loss items overlap with uremic symptoms; low specificity in PD
EFS (Edmonton Frail Scale)	Rolfsen et al. (2006)	17	0–17; Frailty: ≥8	Limited (validated in general elderly; not in PD/CKD)	8–12	Comprehensive (cognitive, functional, nutritional, mood, continence)	Too lengthy for routine clinical use; includes items (e.g., continence) not directly related to frailty in PD
FI (Frailty Index)	Mitnitski & Rockwood (2001)	30–70	0–1; Frailty typically ≥0.25	Yes (validated in CKD and dialysis populations; deficit accumulation model well–suited to multimorbidity)	15–25	Comprehensive; captures multi–system decline.	Time–consuming data collection; requires access to comprehensive medical records; not practical for bedside screening
HALFT	Hoeijmakers et al. (2012)	5	0–5; Cut–off varies	Limited (validated in general hospitalized elderly; not specifically in PD/CKD)	3–5	Quick; focuses on social function, support, and participation.	Social–domain only; misses physical and cognitive frailty; may underestimate frailty in PD patients with significant physical burden
MMSE	Folstein et al. (1975)	30	0–30; Cognitive impairment: <24	Yes (widely used in CKD/PD to screen cognitive impairment, but not a frailty tool)	7–10	Gold standard for cognitive screening; well–validated in PD.	Not a frailty tool—assesses cognition only; cannot capture physical or social frailty; should be combined with other tools
Barthel Index	Mahoney & Barthel (1965)	10	0–100; Dependence: <100	Yes (widely used in CKD/PD to assess functional dependence, but not a frailty tool)	5–10	Simple; widely used by nurses; good for tracking functional decline.	Not a frailty tool—assesses ADL only; ceiling effect (many PD patients score 100); lacks physical, cognitive, and social domains
SPPB	Guralnik et al. (1994)	3	0–12; Low performance: ≤ 9; Frailty proxy: ≤ 8	Yes (validated in CKD and hemodialysis; predicts falls and hospitalization)	5–8	Objective physical performance; predicts adverse outcomes well in CKD.	Requires physical space and patient cooperation; not feasible for bedridden or severely ill PD patients; does not assess cognitive/social domains
OFI−8 (Oral Frailty Index−8)	Nomura et al. (2021)	8	0–8; Cut–off varies	No (developed in community–dwelling elderly; not validated in PD/CKD)	3–5	Quick; addresses under–recognized oral frailty.	Not validated in PD; oral symptoms may be confounded by uremic stomatitis, dry mouth from dialysis, or fluid restriction; narrow scope
CHS (Cardiovascular Health Study criteria)	Fried et al. (2001)	5	0–5; Frailty: ≥3	Yes (same as Fried Phenotype; validated in hemodialysis and early CKD)	10–15	Objective; gold standard; well–validated in CKD.	Same as Fried—requires physical tests; not feasible in bedridden patients; weight loss confounded by fluid overload in PD

## 3 Results

### 3.1 Literature search results

**Figure 1:**
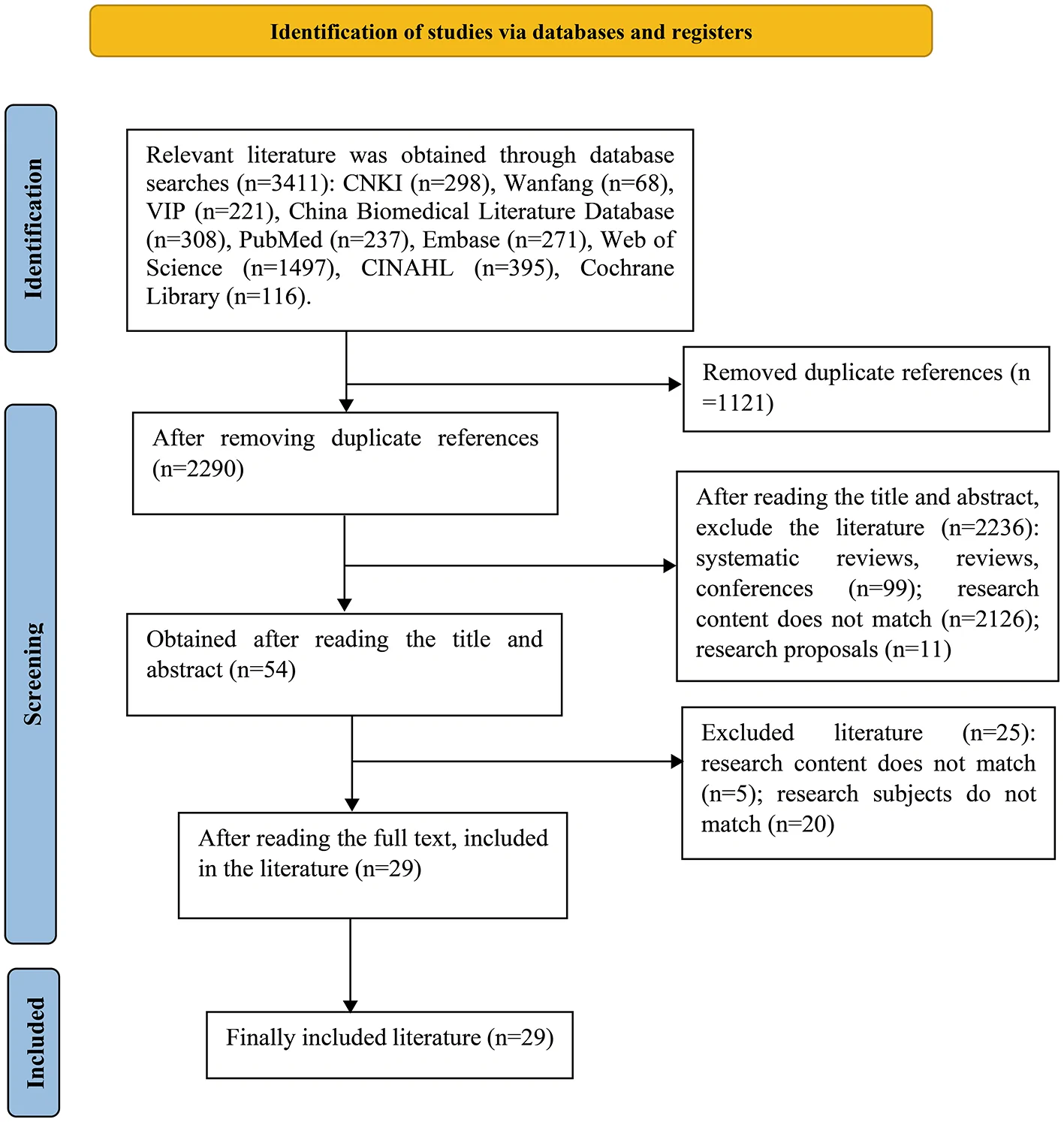
Literature selection process. This figure illustrates the complete process of systematically searching literature across 9 Chinese and English databases. Through rigorous deduplication, initial screening, and full-text review, a total of 29 studies were finally included. This process complies with the PRISMA guidelines.

An initial search of the database retrieved 3,411 articles, and after removing duplicates, 2,290 articles remained. After screening the titles and abstracts, 54 articles remained, and after further full-text review, 29 articles were included. The literature screening process is shown in Figure 1.

### 3.2 Basic characteristics of the included literature

A total of 29 studies were included (2, 3, 11–37), with the basic characteristics shown in Table 2.

### 3.3 Current status of frailty in PD patients

Of the 29 included studies, 28 (2, 3, 11, 12, 14–37) reported the incidence of frailty in PD patients (7.3–83.0%), with considerable variation in incidence across different studies.

### 3.4 Assessment tools for frailty in PD patients

A total of 10 different frailty assessment tools were used in the included studies, with specific information shown in Table 3. According to their measurement purposes, these tools can be divided into those assessing a single dimension of frailty and those involving multidimensional frailty assessment (covering psychological, physiological, and social aspects). The main tools used to assess physical frailty included the Fried Frailty Phenotype Scale (note: the CHS Frailty Scale is the same instrument as the Fried Phenotype, and thus was not counted separately), the Barthel Index (BI), and the Oral Frailty Index-8 (OFI-8). The tools used to assess multidimensional frailty mainly included the Tilburg Frailty Indicator (TFI), the Clinical Frailty Scale (CFS), the Frailty Index (FI), and the Edmonton Frail Scale (EFS). The tools used to assess social frailty were mainly the HALFT Scale and the Short Physical Performance Battery (SPPB), among others. The main tool used to assess cognitive decline was the Mini-Mental State Examination (MMSE), and the rapid screening tool for frailty was primarily the FRAIL Scale. Among the included studies, the most frequently used tools were the Clinical Frailty Scale (CFS, *n* = 8) and the Fried Frailty Phenotype (*n* = 8). It is worth noting that the Barthel Index and the MMSE were used by some authors as instruments to assess functional dependence or cognitive impairment, rather than as frailty-specific tools, and were therefore distinguished in the counting. The specific characteristics of each assessment tool are shown in Table 3.

### 3.5 Critical appraisal of included studies

A total of 29 studies on frailty in patients undergoing peritoneal dialysis (PD) were included in this review. Among these, 20 cross-sectional studies were appraised using the JBI Critical Appraisal Checklist for Analytical Cross-Sectional Studies, eight cohort studies were appraised using the JBI Critical Appraisal Checklist for Cohort Studies, and one mixed-methods study was appraised accordingly. The appraisal results indicated that the overall methodological quality of the included studies was generally high: 16 studies were rated as high quality, 13 as moderate quality, and none as low quality. Cross-sectional studies have limitations such as failure to use objective and standardized diagnostic/assessment criteria, failure to control for confounding factors, and unvalidated exposure measurements or unclear reliability and validity. Cohort studies have limitations such as failure to report strategies for controlling confounding factors, inadequate/incomplete follow-up or unclear assessment of follow-up completeness, and failure to report reasons for loss to follow-up and their handling. Detailed appraisal results are presented in Supplementary Table 2.

### 3.6 Factors affecting frailty in PD patients

Four themes summarizing the factors affecting the frailty of PD patients were identified. (1) Socio-demographic factors: age (3, 11, 14–18, 21, 24, 27, 28, 30, 32–35, 37); gender (35); ethnicity (24); level of education (14, 32, 35); place of residence (14–16); disability (32); employment/unemployment status (32). (2) Disease factors: kidney-related indicators [dialysis age/duration (21, 35); serum creatinine (17, 21, 30, 35); eGFR (35); urine output (21); residual GFR (3, 18); Kt/V (3, 18, 22); pleural effusion (17)]; comorbidity (14, 18, 21, 22, 24, 27, 31, 34, 35, 37) (High or low blood pressure, cardiovascular diseases, diabetes, gout, etc.); Laboratory indicators [Alb (3, 16–18, 20–23, 29, 30, 35), Hb (20), TC (20, 28), TG (20, 35), Ferritin (28), blood potassium (21), iPTH (37)]; Inflammation markers [CRP (20–22, 37), hs-CRP (35)]; CCI (18, 21, 29, 31); Multiple medications (34).(3) Physical function and composition indicators: BMI (14, 21, 32); Body surface area [BSA (30)]; Muscle mass (24); Edema index (21); Mid-Arm Circumference (MAC)/Mid-Arm Muscle Circumference (MAMC) (23); Number of teeth (37); walking speed (37); (4) Psychological and behavioral factors: cognitive function (12, 34); psychological state (depression, loneliness, perceived self-burden) (2, 11, 20, 31, 34); Smoking status (14); alcohol intake (14); sedentary behavior (34); exercise self-efficacy (16); social connections (social isolation, social participation, social support) (11, 15, 34); interpersonal relationships (friend networks, self-disclosure) (11); nutritional status [MIS score (2, 3, 23, 33); malnutrition (13, 34)]; and sleep status (16).

## 4 Discussion

### 4.1 The assessment tools for frailty in PD patients are complex, and there is an urgent need to develop specific assessment tools

This study systematically reviewed the assessment tools for frailty in PD patients, providing a basis for clinical selection, but also revealed many methodological limitations of existing tools, which need to be optimized in the future. (1) Disease-specific issues, as the frailty assessment tools used in the included studies (such as OFI-8,CFS,HALFT,EFS, and TFI) are mostly general scales, lacking validation in patients with PD. In terms of assessment content, existing tools lack specificity for dialysis-related factors unique to PD patients, making it difficult to fully reflect the impact of fluid load, dialysis adequacy, microinflammatory status, and peritonitis on the occurrence and development of frailty. Therefore, in the future, assessment tools specific to peritoneal dialysis should be developed and validated based on the disease characteristics of PD patients, incorporating more dialysis-related sensitive indicators to improve the specificity and accuracy of the assessment. (2) The timing of assessment remains to be clarified. Although studies on frailty in PD patients are gradually increasing, most relevant studies (2, 18, 20, 33) both domestically and internationally have not clearly specified the timing of assessment, which may lead to considerable variability in results. Future research urgently needs to clarify the baseline timing and key time points for frailty assessment in PD patients, in order to establish a standardized assessment protocol. (3) There is a lack of dynamic assessment and longitudinal follow-up. Studies in this field are predominantly cross-sectional surveys, making it difficult to continuously measure frailty over time. Given the increasing dialysis vintage and the occurrence of complications in PD patients, this assessment model limits in-depth exploration of the trajectory of frailty development, its influencing factors, and optimal timing for intervention (4). Cultural applicability needs to be strengthened. The validation populations for some tools (such as the FRAIL,EFS,CHS) are largely limited to Western countries, lacking large-sample, multicenter application evidence in Asian populations, particularly in the Chinese population. Dong et al. (38) conducted a reliability and validity test of the Chinese version of the TFI among 917 community-dwelling older adults in China. The results showed that the Cronbach's α coefficient of the total scale was 0.71, indicating an acceptable level of internal consistency among items; the test-retest reliability coefficient was *r* = 0.88, suggesting good temporal stability of the scale. Cai (39)evaluated the reliability and validity of the FI in Chinese hospitalized older patients, reporting a Cronbach's α coefficient of 0.874 and a test-retest reliability coefficient of 0.948, demonstrating that the scale possesses good internal consistency and temporal stability in this population. In summary, the TFI and FI have demonstrated satisfactory reliability and validity in Chinese populations. Future research should conduct large-sample, multicenter studies based on the disease-specific characteristics of PD patients, with the aim of establishing a standardized frailty assessment tool tailored to the Chinese PD patients.

### 4.2 The incidence of frailty varies considerably among PD patients and is influenced by multiple factors, necessitating comprehensive assessment in clinical intervention

This study found that the prevalence of frailty in PD patients ranged from 7.3% to 83%, exhibiting considerable heterogeneity. This variation may be attributed to the following factors: (1) Methodological differences in assessment tools. Different frailty assessment tools, varying in dimensional emphasis and cutoff thresholds, significantly influenced the reported prevalence rates. Studies employing the Clinical Frailty Scale (CFS) [e.g., Chan et al. (2) reported 72.7%, Ng et al. (3) reported 69.4%] tended to yield higher prevalence rates, as CFS relies on clinicians' subjective global judgment and is more sensitive to functional dependence and comorbidities. In contrast, studies using the Fried Frailty Phenotype [e.g., Zhang et al. (16) reported 24.3%] were strictly based on objective physiological indicators (e.g., grip strength, gait speed) and produced relatively lower prevalence rates. Brief screening tools, such as the FRAIL Scale, tended to underestimate frailty prevalence [e.g., Du et al. (22) reported only 12.62%], owing to their limited number of items and lack of assessment in cognitive and social dimensions. (2) Regional and ethnic background differences reflected the impact of healthcare accessibility and socioeconomic factors. This review indicated that studies from Asia, particularly China, generally reported higher frailty prevalence rates (ranging from 24.1% to 76.6%), whereas those from Europe [e.g., Davenport (24) reported 7.3%, Mansur et al. (26) reported 47.8%] and Japan [Kamijo et al. (25) reported 10.9%; Kobayashi et al. (37) reported 29.4%] reported relatively lower rates. This regional disparity suggests that, beyond ethnic differences, variations in national healthcare reimbursement policies, dialysis center management quality, and public health literacy may affect volume management, nutritional status, and complication control in PD patients, thereby altering the trajectory of frailty development. (3) Differences in the patient populations enrolled across studies might also contribute to the variability in prevalence rates. Studies predominantly comprising elderly patients [e.g., Zhu et al. (20), with a higher mean age, reported a prevalence of 74.14%] showed significantly higher rates than those including younger dialysis populations. However, given that the Barthel Index measures functional dependence rather than frailty *per se*, this estimate should be interpreted with caution. Chen et al. (21) indicated that patients with longer dialysis vintage bore a heavier volume load and faced a higher risk of frailty. Studies conducted in China (3, 14, 18–20, 27, 31, 33)have consistently demonstrated a relatively high prevalence of frailty in PD patients, warranting greater clinical attention from healthcare providers.

Currently, frailty in PD patients is jointly influenced by multidimensional factors, including sociodemographic, disease-related, body composition indicators, and psychological-behavioral factors. Therefore, clinical intervention requires a comprehensive assessment. (1) Sociodemographic factors: Advanced age is a risk factor for frailty in PD patients, which may be attributed to white matter lesions, gray matter loss, reduced dendritic branching, and decreased dopaminergic activity (35). Additionally, as patients age, they become more susceptible to malnutrition, decreased muscle mass, and reduced immunity, all of which contribute to the occurrence of frailty (40). Patients with higher education levels have a lower incidence of frailty, possibly because they are better able to understand and accept health education provided by healthcare professionals, leading to higher adherence (32). Both unemployment and disability are risk factors for frailty, possibly because they can lead to reduced economic income and decreased physical activity, which in turn affect nutritional status and accelerate muscle atrophy;At the same time, they can also lead to social isolation and trigger social frailty (32). In some studies, no significant differences were observed in factors such as gender or race, suggesting that the impact of sociodemographic factors may vary depending on sample characteristics or cultural background. Prospective studies are needed to further validate these findings. (2) Disease-related factors: Kidney-related indicators. Dialysis-related parameters such as longer dialysis vintage, decline in residual renal function (including residual GFR and urine output), and inadequate dialysis adequacy (Kt/V) can accelerate protein-energy wasting and the accumulation of uremic toxins, thereby promoting the development of frailty (3, 17). Serological indicators. Low albumin and low hemoglobin reflect malnutrition and anemia, while elevated CRP/hs-CRP indicates microinflammatory activation. Together, these three factors constitute the connection within the “malnutrition-inflammation-frailty” axis. Comorbidity burden is critically important. PD patients often have comorbidities such as cardiovascular disease, diabetes, and unstable blood pressure, which exacerbate symptom burden and are closely associated with the development of frailty. Persistent protein-energy wasting (PEW), resulting from inadequate dietary intake, protein loss in dialysate, and uremic metabolic disturbances, depletes the body's protein reserves and reduces muscle mass, which in turn directly leads to decreased handgrip strength and impaired physical function, thereby manifesting as frailty (41). Concurrently, bioincompatible dialysis solutions, endotoxin translocation resulting from impaired intestinal barrier function, and the accumulation of advanced glycation end-products (AGEs) collectively trigger a state of persistent, low-grade chronic inflammation. This inflammatory state promotes skeletal muscle catabolism via the ubiquitin-proteasome pathway and inhibits albumin synthesis, thereby establishing a sustained catabolic vicious cycle. Notably, the aforementioned two processes are not mutually independent: hypoalbuminemia compromises the body's antioxidant capacity and immune function, rendering patients more susceptible to inflammatory stimuli; conversely, proinflammatory cytokines (e.g., IL-6, TNF-α) not only facilitate muscle wasting but also further suppress appetite, thereby exacerbating nutritional depletion (42). Furthermore, the excessive production of reactive oxygen species (ROS) in the uremic milieu can directly induce mitochondrial dysfunction in skeletal muscle cells, leading to reduced ATP generation and premature muscle fatigue—a phenomenon clinically manifested as the most prominent fatigue and muscle strength decline in PD patients. Frailty in PD patients cannot be adequately explained by any single factor; rather, it arises from the synergistic interplay among the metabolic, nutritional, and inflammatory disturbances that are uniquely characteristic of this patient population. Future research should conduct multicenter longitudinal studies targeting modifiable indicators (e.g., improving dialysis adequacy, preserving residual renal function), serological indicators (e.g., correcting hypoalbuminemia and anemia, anti-inflammatory therapy), and comorbidity management to validate causal relationships and develop precise intervention strategies. (3) Body composition indicators: Frailty may be a condition characterized by low muscle mass and high fat, and is closely related to nutritional status. A BMI that is too high or too low can lead to frailty (43). MAC, MAMC, and BSA reflect the nutritional status of patients, and if too low, can lead to frailty in PD patients (23, 30). A higher oedema index is associated with muscle loss and malnutrition, which can lead to frailty (44). Healthcare professionals should pay attention to patients‘ nutritional status and muscle mass, and regularly monitor patients' body indices. A reduction in teeth can affect chewing function, leading to insufficient food intake, indirectly causing malnutrition and accelerating weakness (37). For every 1 m/s decrease in walking speed, the risk of frailty increases, which may be due to the slowing of walking speed reflecting a decline in lower limb strength, leading to limited mobility, subsequently causing muscle atrophy and social isolation, ultimately accelerating the onset of frailty (37). Future research should conduct longitudinal cohort studies to regularly monitor body composition indicators, oral health status, and gait speed in peritoneal dialysis patients, and based on these findings, develop individualized nutritional support and exercise rehabilitation intervention programs to delay or prevent the onset of frailty. (4) Psychological and behavioral factors: Psychological aspects: The greater the sense of loneliness and social isolation, the lower the friend's network score and the more severe the degree of social frailty (11). The prevalence of social function impairment in PD patients is 70%, and their loneliness scores are higher than those of patients with other chronic diseases, possibly due to the distance and time constraints of multiple dialysis sessions limiting social interaction (45). Moreover, when PD patients experience cognitive decline that further impairs physical health, it can exacerbate their sense of loneliness and increase the risk of frailty (34). Behavioral level: MIS score is positively correlated with frailty score (33). A high MIS score indicates that the patient is suffering from both malnutrition and chronic inflammatory burden, which interact with each other, directly impairing the body's muscle synthesis and physiological reserve capacity, leading to and exacerbating frailty. Smoking and drinking are independent risk factors for frailty in patients with PD (14). Because carbon monoxide inhaled from smoke forms carboxyhaemoglobin in the body, further reducing the oxygen-carrying capacity of hemoglobin, it easily causes cerebral hypoxia, dizziness, memory decline, and brain dysfunction, thereby leading to frailty. Long-term alcohol consumption can suppress central nervous system function, reduce balance, muscle strength and motor coordination, and increase the risk of falling. Low exercise self-efficacy and sedentary behavior are very common among dialysis patients, which can increase the risk of cardiovascular events and lead to problems such as decreased muscle mass, contributing to the onset of frailty (16, 34). Insomnia is a risk factor for frailty in PD patients, as it easily leads to the activation of inflammatory factors, reduced protein synthesis, decreased immunity, and subsequently, the occurrence of frailty (16). Future research should develop comprehensive psychosocial support and behavioral intervention programs for peritoneal dialysis patients, and verify their effectiveness in reducing the risk of frailty through randomized controlled trials (RCTs).

In summary, medical staff should systematically assess multidimensional factors and establish multidisciplinary teams in psychology, rehabilitation, nutrition, and other fields to more comprehensively prevent and treat frailty in PD patients.

### 4.3 Research on frailty in PD patients continues to develop, and future studies should delve deeper

Currently, research on frailty in PD patients is showing a continuous development trend, with a gradual increase in both domestic and international literature, indicating that this topic is increasingly becoming a significant public health issue. Future research can be further explored from the following four directions to promote evidence accumulation and clinical translation in this field: (1) Develop and validate PD-specific frailty assessment tools. Most existing tools originate from community-dwelling older adults or hemodialysis populations and have not fully accounted for the unique clinical characteristics of PD therapy. Future research should focus on developing or validating frailty assessment tools specifically tailored to the PD population, incorporating dialysis-related parameters such as dialysis adequacy, volume overload status, and history of peritonitis into the assessment framework. Furthermore, cross-cultural adaptation and reliability/validity testing should be conducted across patients from different geographical regions, disease stages, and demographic characteristics to ensure fairness and dynamic applicability. (2) Conduct multicenter longitudinal cohort studies to identify modifiable risk factors. The majority of current studies are cross-sectional in design, which cannot establish the temporal sequence or causal relationships between frailty and various factors. Future research should carry out large-sample, multicenter prospective longitudinal cohort studies with long-term follow-up of PD patients, systematically evaluate the dynamic trajectory of frailty over time, and identify modifiable risk factors (e.g., nutritional status, inflammatory levels, physical activity, psychological status), thereby providing causal evidence for the development of intervention strategies. (3) Establish standardized time points for frailty assessment. There is currently a lack of uniform standards regarding the timing of frailty assessment across studies, which limits the comparability of results between studies. It is recommended that future research establish a unified assessment time framework during PD follow-up, such as at baseline (initiation of PD), at 6 months, at 12 months, and annually thereafter. A standardized assessment protocol will facilitate the establishment of longitudinal databases, enable cross-study comparisons, and help standardize clinical screening procedures. (4) Conduct randomized controlled intervention studies targeting PD patients. Existing evidence is primarily focused on the association between frailty and various factors, with relatively few interventional studies on frailty in PD patients. Future research should design and implement high-quality randomized controlled trials to evaluate the effectiveness of comprehensive intervention programs tailored to the characteristics of PD patients—including multicomponent interventions such as nutritional support, exercise rehabilitation training, and psychosocial support—on improving frailty status, thereby providing high-level evidence for clinical practice.

### 4.4 Strengths and limitations

This study employed a scoping review methodology to systematically synthesize the prevalence of frailty in peritoneal dialysis patients, the characteristics and limitations of assessment tools, and the identified influencing factors across 29 included studies. It also identified research gaps in this field, providing clear direction for future development of PD-specific frailty assessment tools, conduct of longitudinal follow-up studies, and construction of precise intervention strategies.

In addition, this study has the following limitations. (1) The prevalence of frailty reported across the included studies exhibited substantial heterogeneity (ranging from 7.3% to 83%). Although we conducted qualitative stratified analyses in the discussion section across three dimensions—assessment tools, geographical distribution, and clinical characteristics (age, dialysis vintage, and comorbidity burden)—we were unable to quantitatively estimate the contribution of each factor to the observed heterogeneity through methods such as meta-regression. This was due to the predominance of cross-sectional designs among the included studies, the lack of individual-level raw data, and the fact that most studies did not pre-specify subgroup analyses by key strata (e.g., age groups or dialysis vintage categories). (2) The majority of the included studies were cross-sectional in design, with a relatively limited number of prospective cohort studies available. Therefore, the factors identified in this review should be interpreted as associated factors rather than causal determinants, as the temporal sequence and direction of causality cannot be established based on the current evidence. Future research with longitudinal designs is warranted to clarify causal relationships. (3) Of the 29 studies included in this review, the majority originated from China (*n* = 21), with additional studies from Portugal (*n* = 2), Japan (*n* = 2), South Korea (*n* = 1), the United Kingdom (*n* = 1), Chile (*n* = 1), and Mexico (*n* = 1). This geographical concentration limits the generalizability of our findings to other populations and healthcare settings. Caution should therefore be exercised when extrapolating these conclusions to regions with different healthcare systems, ethnic compositions, or socioeconomic contexts, and future cross-cultural, multinational studies are warranted to validate the consistency of these findings. (4) The causal relationships between the identified risk factors and frailty cannot be determined from this review. Although we systematically summarized the associations between multidimensional variables—including age, dialysis vintage, nutritional status, inflammatory markers, physical function, and psychosocial factors—and frailty in the results section, the vast majority of the included studies were cross-sectional in design, which can only reflect correlations at a single time point and are unable to track the dynamic trajectory of frailty status over time. Even though some prospective cohort studies have suggested the predictive value of certain factors for the development of frailty, the substantial heterogeneity across studies in terms of assessment tools, follow-up time points, and outcome definitions, combined with the fact that most studies did not adequately control for potential time-dependent confounding factors (e.g., adjustments in dialysis regimens, occurrence of comorbidities), means that the current evidence base is insufficient to support causal inference.(5) It should be noted that there may be potential overlap between the included cohorts. Four of the included studies (2, 3, 31, 33) originated from the same research center in Hong Kong, two of which (2, 31) reported a sample size of 267. Although these studies addressed distinct research questions, the possibility of overlapping patient populations cannot be entirely excluded. This should be considered when interpreting the pooled descriptive findings.

Consequently, the synthesized findings of this review should be interpreted as primarily reflecting the current state of research on frailty in PD patients in China and other selected countries, with limited representation from other regions, and caution should be exercised when extrapolating these conclusions to other regions. Future studies should prioritize cross-cultural, multinational collaborative research to validate the consistency of these findings across different geographical areas and healthcare systems.

### 4.5 Implications for clinical practice

The incidence of frailty in PD patients ranges from 7.3% to 83%. Clinical care should incorporate frailty assessment into routine follow-up, and it is recommended to use the Fried frailty phenotype scale or the Clinical Frailty Scale (CFS) for systematic screening to identify high-risk groups early. Nursing strategies should consider influencing factors such as age, nutritional status and exercise self-efficacy, and include: Nutritional management: monitor serum albumin and guide intake of high-quality protein; Exercise rehabilitation: develop individualized exercise programmes based on guidelines and expert consensus; Sleep and psychological support: improve insomnia and pay attention to cognitive function. Implement full-cycle rehabilitation care through MDT collaboration to prevent falls and other adverse events and improve patients' quality of life. At the implementation level, it is recommended that frailty assessment be integrated into routine multidisciplinary team-based care, with clear role delineation among team members: nephrologists are responsible for incorporating frailty screening into comprehensive assessments at the initiation of peritoneal dialysis (PD) and during regular follow-up visits, interpreting the results in conjunction with dialysis adequacy, volume status, and comorbidities; dialysis specialty nurses administer the assessment tools during monthly follow-up appointments or PD fluid exchange training sessions, accurately document the results, and provide feedback to the team; dietitians conduct dietary surveys for patients who screen positive and develop individualized nutritional support plans; rehabilitation therapists design feasible exercise prescriptions (e.g., aerobic training, resistance training, and balance training) tailored to the patient's frailty severity and baseline physical capacity; and psychologists identify and intervene in psychological issues such as depression, anxiety, or insufficient social support, providing appropriate interventions when necessary. Additionally, it is recommended to establish a standardized “screening-assessment-intervention-follow-up” clinical pathway in the PD outpatient clinic, with clearly defined assessment time points (e.g., baseline assessment, reassessment every 6 months, and reassessment following adverse events), and to convene regular case discussion meetings to dynamically adjust management strategies.

## 5 Summary statement

This article systematically reviews the current status, assessment tools, and influencing factors of frailty in PD patients, and nursing staff can choose precise assessment scales based on specific clinical situations and research purposes. Future research can further develop localized frailty assessment tools that align with our cultural background and are tailored to the characteristics of PD patients. Frailty is influenced by multiple factors such as nutrition, inflammation, and psychology. Nursing interventions should not be limited to a single dimension; an integrated plan of ‘nutritional support, exercise, and psychological counselling' can be developed, while also mobilizing family and social support systems to effectively prevent and reduce frailty in PD patients, improve their prognosis, and enhance quality of life.

## Data Availability

The original contributions presented in the study are included in the article/Supplementary material, further inquiries can be directed to the corresponding authors.
