# Supplementary material for: Frailty in peritoneal dialysis patients: a scoping review
Source: Front Med (Lausanne). 2026 Aug 20;13:1906681. doi: 10.3389/fmed.2026.1906681 (PMC13539128; doi:10.3389/fmed.2026.1906681)
Supplement: Supplementary file 1 [file Supplementary_file_1.pdf]

S1 File. Search strategy for all databa

CNKI：(SU=腹膜透析 OR SU=透析 OR SU=终末期肾病 OR SU=ESRD) AND (SU=衰弱 OR SU=虚弱 OR SU=衰弱综合征 OR SU=社会衰弱 OR SU=躯体衰弱 OR SU=心理衰弱 OR SU=认知衰弱)

WanFang ：(主题:("腹膜透析" OR "透析" OR "终末期肾病" OR "ESRD")) AND (主题:("衰弱" OR "虚弱" OR "衰弱综合征" OR "社会衰弱" OR "躯体衰弱" OR "心理衰弱" OR "认知衰弱"))

VIP：(T=(腹膜透析 OR 透析 OR 终末期肾病 OR ESRD)) AND (T=(衰弱 OR 虚弱 OR 衰弱综合征 OR 社会衰弱 OR 躯体衰弱 OR 心理衰弱 OR 认知衰弱))

China Biomedical Literature Database ：("腹膜透析"[常用字段] OR "透析"[常用字段] OR "终末期肾病"[常用字段] OR "ESRD"[常用字段]) AND ("衰弱"[常用字段] OR "虚弱"[常用字段] OR "衰弱综合征"[常用字段] OR "社会衰弱"[常用字段] OR "躯体衰弱"[常用字段] OR "心理衰弱"[常用字段] OR "认知衰弱"[常用字段])
