# Supplementary material for: Frailty in peritoneal dialysis patients: a scoping review
Source: Front Med (Lausanne). 2026 Aug 20;13:1906681. doi: 10.3389/fmed.2026.1906681 (PMC13539128; doi:10.3389/fmed.2026.1906681)
Supplement: Supplementary file 2 [file Supplementary_file_2.pdf]

JBI Quality Appraisal for Quantitative Studies

Included Studies	1. Were the inclusion criteria for the sample clearly defined?	2. Were the study subjects and setting described in detail?	3. Was the exposure measured in a valid and reliable way?	4. Were objective, standardized criteria used to measure the condition?	5. Were confounding factors identified?	6. Were strategies for controlling confounding factors stated?	7. Were the outcome measures valid and reliable?	8. Was an appropriate statistical analysis used?	Overall Quality Rating
Peng et al.	Yes	Yes	Yes	No	Yes	Yes	Yes	Yes	Moderate quality
Wang et al	Yes	No	Yes	Yes	Yes	Yes	Yes	No	Moderate quality
Xie et al	Yes	Yes	Yes	Yes	No	No	Yes	No	Moderate quality
Yao et al	Yes	Yes	No	Yes	Yes	Yes	Yes	No	Moderate quality
Yi et al	Yes	Yes	Yes	No	Yes	Yes	Yes	No	Moderate quality
Zhang et al	Yes	Yes	Yes	Yes	Yes	Yes	Yes	Yes	High quality
Li et al	No	Yes	Yes	Yes	Yes	Yes	Yes	Yes	High quality
Zhu et al	Yes	Yes	Yes	No	Yes	Yes	No	Yes	Moderate quality
Hu et al	Yes	Yes	Yes	Yes	不清楚	Yes	Yes	Yes	High quality
Zhu et al	Yes	No	Yes	Yes	Yes	Yes	Yes	No	Moderate quality
Chen et al	Yes	Yes	Yes	Yes	Yes	Yes	Yes	Yes	High quality
Du et al	Yes	Yes	No	Yes	Yes	Yes	No	Yes	Moderate

Included Studies	1. Were the inclusion criteria for the sample clearly defined?	2. Were the study subjects and setting described in detail?	3. Was the exposure measured in a valid and reliable way?	4. Were objective, standardized criteria used to measure the condition?	5. Were confounding factors identified?	6. Were strategies for controlling confounding factors stated?	7. Were the outcome measures valid and reliable?	8. Was an appropriate statistical analysis used?	Overall Quality Rating
									quality
Meng et al	Yes	Yes	Yes	Yes	Yes	Yes	Yes	No	High quality
Davenport et al	Yes	Yes	Yes	Yes	Yes	Yes	Yes	Yes	High quality
Mansur et al	Yes	Yes	Yes	Yes	Yes	Yes	Yes	Yes	High quality
Liu et al	Yes	Yes	Yes	No	Yes	Yes	Yes	No	Moderate quality
Torres et al	Yes	Yes	Yes	No	Yes	No	Yes	No	Moderate quality
Chun-Kau et al	Yes	Yes	Yes	Yes	Yes	Yes	Yes	Yes	High quality
NoPeralta-Flores et al	Yes	Yes	Yes	No	Yes	No	Yes	No	Moderate quality
Ng et al	Yes	Yes	Yes	No	Yes	Yes	Yes	Yes	High quality

JBI Critical Appraisal Checklist for Cohort Studies

Included Studies	1. Were two comparable groups selected based on similar populations and grouped at the same time point?	2. Was the exposure measured using similar methods?	3. Was the outcome measured using similar methods?	4. Were confounding factors identified?	5. Were strategies for controlling confounding factors stated?	6. Were participants free of the outcome event at baseline?	7. Was follow-up adequate and complete?	8. Were valid and reliable methods used to assess the outcome?	9. Was the follow-up duration long enough to observe the occurrence of outcomes?	10. Were reasons for loss to follow-up and their handling reported?	11. Was an appropriate statistical analysis used?	Overall Quality Rating
Chanet al	Yes	Yes	Yes	Yes	Yes	Yes	No	Yes	Yes	No	No	Moderate quality
Kamijo et al	Yes	Yes	Yes	Yes	No	Yes	Yes	Yes	Yes	Yes	Yes	High quality
Chen et al	Yes	Yes	Yes	Yes	Yes	Yes	Yes	Yes	Yes	Yes	Yes	High quality
Piedade et al	Yes	Yes	Yes	Yes	Yes	Yes	Unclear	Yes	NA	NA	Yes	Moderate quality
Gordon et al	Yes	Yes	Yes	Yes	Yes	Yes	Yes	Yes	Yes	Yes	Yes	High quality
Lee et al	Yes	Yes	Yes	Yes	Yes	Yes	Yes	Yes	Yes	Yes	Yes	High quality

Included Studies	1. Were two comparable groups selected based on similar populations and grouped at the same time point?	2. Was the exposure measured using similar methods?	3. Was the outcome measured using similar methods?	4. Were confounding factors identified?	5. Were strategies for controlling confounding factors stated?	6. Were participants free of the outcome event at baseline?	7. Was follow-up adequate and complete?	8. Were valid and reliable methods used to assess the outcome?	9. Was the follow-up duration long enough to observe the occurrence of outcomes?	10. Were reasons for loss to follow-up and their handling reported?	11. Was an appropriate statistical analysis used?	Overall Quality Rating
Yi et al	Yes	Yes	Yes	Yes	Yes	Yes	Yes	Yes	Yes	Yes	Yes	High quality
Kobayashi et al	Yes	Yes	Yes	Yes	Yes	Yes	No	Yes	Yes	No	Yes	High quality

JBI Critical Appraisal Checklist for Mixed Methods Research

Included Studies	1. Were the inclusion criteria for the sample	2. Were the study subjects and	3. Was the exposure measured	4. Were objective, standardized criteria used	5. Were confounding factors identified?	6. Were strategies for controlling	7. Were the outcome measures	8. Was an appropriate statistical analysis	Overall Quality Rating

	clearly defined?	setting described in detail？	in a valid and reliable way?	to measure the condition?		confounding factors stated?	valid and reliable?	used?	
Cao et al	Yes	Yes	Yes	Yes	Yes	Yes	Yes	Yes	High quality

Included Studies	1. Were two comparable groups selected based on similar populations and grouped at the same time point?	2. Was the exposure measured using similar methods?	3. Was the outcome measured using similar methods?	4. Were confounding factors identified?	5. Were strategies for controlling confounding factors stated?	6. Were participants free of the outcome event at baseline?	7. Was follow-up adequate and complete?	8. Were valid and reliable methods used to assess the outcome?	9. Was the follow-up duration long enough to observe the occurrence of outcomes?	10. Were reasons for loss to follow-up and their handling reported?	11. Was an appropriate statistical analysis used?	Overall Quality Rating
Cao et al	Yes	Yes	Yes	Yes	Yes	Yes	Yes	Yes	Yes	Yes	Yes	High quality
