# Supplementary material for: Frailty in peritoneal dialysis patients: a scoping review
Source: Front Med (Lausanne). 2026 Aug 20;13:1906681. doi: 10.3389/fmed.2026.1906681 (PMC13539128; doi:10.3389/fmed.2026.1906681)
Supplement: Supplementary file 3 [file Supplementary_file_3.pdf]

PRISMA-ScR

No.	PRISMA-ScR items	Location in This Study (Section Heading)
1	Identify the report as a scoping review.	Title page
2	Provide a structured summary that includes (as appl icable): background, objectives, eligibility criteria, s ources of evidence, charting methods, results, and c onclusions that relate to the review questions and o bjectives.	Abstract
3	Describe the rationale for the review in the context of what is already known. Explain why the revie w questions/objectives lend themselves to a scoping review approach	1 Introduction
4	Provide an explicit statement of the questions and objectives being addressed with reference to their k ey elements (e.g., population or participants, concep ts, and context) or other relevant key elements used to conceptualize the review questions and/or object ives.	2.1 Identifying the Research Question
5	Indicate whether a review protocol exists; state if a nd where it can be accessed (e.g., a Web address); and if available, provide registration information, i ncluding the registration number.	1 Introduction
6	Specify characteristics of the sources of evidence u sed as eligibility criteria (e.g., years considered, lan guage, and publication status), and provide a ration ale.	2.2 Inclusion and Exclusion C riteria
7	Describe all information sources in the search (e.g., databases with dates of coverage and contact with authors to identify additional sources), as well as t he date the most recent search was executed.	2.3 Search Strategy
8	Present the full electronic search strategy for at lea st 1 database, including any limits used, such that i t could be repeated.	Table 1
9	State the process for selecting sources of evidence (i.e., screening and eligibility) included in the scopi ng review.	2.4 Literature Screening and Data Extraction and 3.1 Liter ature Search Results
10	Describe the methods of charting data from the inc luded sources of evidence (e.g., calibrated forms or forms that have been tested by the team before th eir use, and whether data charting was done indepe ndently or in duplicate) and any processes for obtai ning and confirming data from investigators.	2.4 Literature Screening and Data Extraction

11	List and define all variables for which data were sought and any assumptions and simplifications made.	2.4 Literature Screening and Data Extraction
12	If done, provide a rationale for conducting a critical appraisal of included sources of evidence; describe the methods used and how this information was used in any data synthesis (if appropriate).	3.5 Critical Appraisal of Included Studies
13	Describe the methods of handling and summarizing the data that were charted.	2.5 Collate, summarize, and report the results
14	Give numbers of sources of evidence screened, assessed for eligibility, and included in the review, with reasons for exclusions at each stage, ideally using a flow diagram.	3.1 Literature Search Results (including Figure 1)
15	For each source of evidence, present characteristics for which data were charted and provide the citations.	Table 2 (and accompanying text in 3.2 Basic characteristics)
16	If done, present data on critical appraisal of included sources of evidence (see item 12).	3.5 Critical Appraisal of Included Studies (and Supplementary Table 2)
17	For each included source of evidence, present the relevant data that were charted that relate to the review questions and objectives.	Table 2 and Table 3 (presented in sections 3.2 and 3.4)
18	Summarize and/or present the charting results as they relate to the review questions and objectives.	Sections 3.3, 3.4, and 3.6 (Current status, Assessment tools, and Influencing factors)
19	Summarize the main results (including an overview of concepts, themes, and types of evidence available), link to the review questions and objectives, and consider the relevance to key groups.	Section 4. Discussion (overall) and 4.5 Implications for Clinical Practice
20	Discuss the limitations of the scoping review process.	4.4 Strengths and Limitations
21	Provide a general interpretation of the results with respect to the review questions and objectives, as well as potential implications and/or next steps.	5. Summary Statement and 4.3 Research on frailty in PD patients continues to develop, and future studies should delve deeper
22	Describe sources of funding for the included sources of evidence, as well as sources of funding for the scoping review. Describe the role of the funders of the scoping review.	Funding Statement (at the end of the manuscript)
